# Synergistic Virus Neutralizing Activities of European Black Elderberry Fruit Extract and Iota-Carrageenan Against SARS-CoV-2, Influenza A Virus and Respiratory Syncytial Virus

**DOI:** 10.3390/nu18081205

**Published:** 2026-04-10

**Authors:** Christian Setz, Melanie Setz, Pia Rauch, Oskar Schleicher, Stephan Plattner, Andreas Grassauer, Ulrich Schubert

**Affiliations:** 1 Harald zur Hausen Institute of Virology, Uniklinikum Erlangen, Friedrich-Alexander University Erlangen-Nürnberg (FAU), 91054 Erlangen, Germany; christian.setz@uk-erlangen.de (C.S.); melanie.setz@uk-erlangen.de (M.S.); pia.rauch@uk-erlangen.de (P.R.); oskarschleicher01@googlemail.com (O.S.); 2 Iprona Lana SpA, Industriestraße 1/6, I-39011 Lana, Italy; stephan.plattner@iprona.com; 3 Marinomed Biotech AG, A-2100 Korneuburg, Austria; andreas.grassauer@marinomed.com

**Keywords:** European black elderberry fruit extract, iota-carrageenan, natural substance, anthocyanins, phenolic compounds, sulfated polymer, SARS-CoV-2, influenza A virus, IAV, respiratory syncytial virus (RSV), antiviral, pandemic preparedness, virus neutralization

## Abstract

**Background/Objectives:** Seasonal waves of respiratory viruses—including SARS-CoV-2, influenza A virus (IAV), and respiratory syncytial virus (RSV)—continue to pose a global health burden and highlight the need for antiviral agents that are effective, safe, broadly active, affordable, and widely accessible. Current interventions are limited by the need for their early administration, the risk of resistance, their costs, and the restricted availability in large parts of the world. For certain natural products, such as European black elderberry (*Sambucus nigra* L.) fruit extract (ElderCraft^®^; EC) and the seaweed-derived sulfated polymer iota-carrageenan (IC), antiviral activities against respiratory viruses, particularly IAV and SARS-CoV-2, have previously been shown. Here, we assessed the antiviral activity of IC and an anthocyanin-standardized EC extract against SARS-CoV-2, IAV, and RSV, either as monotherapy or in multiple-dose combinations. **Methods:** MDCKII cells were infected with IAV_PR8_, human Calu-3 lung epithelial cells with the SARS-CoV-2 Omicron variant, and HEp-2 cells with RSV (A2 strain). Inhibitors were administered either by pre-incubation of cell-free virions prior to infection or, in separate time-of-addition experiments, during or post-infection. Viral replication was quantified by qRT-PCR or intracellular immunostaining. Cytotoxicity was evaluated using a neutral red uptake assay. **Results:** Most intriguingly, both EC and IC are able to neutralize virions derived from SARS-CoV-2, IAV, or RSV extracellularly in a dose-dependent manner. Notably, EC and IC alone exhibited strong anti-RSV activity, which was not reported previously. Most importantly, combined treatment with IC and EC caused a pronounced synergistic antiviral effect against the tested viruses, as confirmed by the Bliss independence model, without any detectable impact on cell viability. Finally, solutions prepared from matrix-standardized mono- or combi-lozenges, containing IC and/or EC in high or low doses, reproduced the antiviral and synergistic combination effects observed with the pure compounds. **Conclusions:** In summary, these findings support further development of EC and IC as a topically accessible, virion-neutralizing combination (e.g., lozenges) to provide additional protection against major respiratory viruses and potentially strengthen pandemic preparedness.

## 1. Introduction

Even after the shift from pandemic spread, which has altogether caused 779 million cases and over 7 million deaths (https://covid19.who.int/ (accessed on 4 February 2026)), to endemic circulation, SARS-CoV-2 has now adapted to humans as a novel respiratory virus. Driven by the ongoing emergence of variants of concern (VoCs), this novel respiratory virus continues to represent a major public health burden. Witnessing the two major pandemic outbreaks of SARS (2002) and SARS-CoV-2 (2019) underlines the urgent need for pandemic preparedness for newly emerging and/or re-emerging viruses [1]. Multiple effective vaccines against SARS-CoV-2 infection have been developed and approved worldwide. However, sterilizing immunity that would prevent SARS-CoV-2 transmission and thereby limit viral adaptation to the human population has not been achieved [2,3]. Although specific antiviral therapies like Paxlovid^®^ and molnupiravir are available, in most cases, clinical benefit was only detected for early-initiated treatment protocols. In addition, broadly applicable therapeutic and prophylactic agents with minimal adverse effects and affordable costs remain limited [4]. Furthermore, COVID-19 and long/post-COVID conditions remain a serious and so far unresolved public health issue, in particular for patients facing a high risk for the development of severe COVID-19, including those aged ≥65 years, and patients with comorbidities, including cardiovascular diseases, lung diseases, diabetes, cancer, obesity, or immunosuppression [5]. Facing the potential of future pandemic events, there remains an unmet medical need for antiviral agents targeting SARS-CoV-2 and other seasonal respiratory viruses that are well tolerated, widely accessible, broad in activity, inexpensive, and rapidly accessible, ideally over the counter.

Comparable to SARS-CoV-2, influenza A virus (IAV) represents another highly contagious respiratory virus that causes seasonal epidemics with substantial morbidity and mortality worldwide. It is estimated that approximately 10% of the world’s population becomes infected, causing approximately 500,000 deaths each year [6]. Influenza commonly goes along with fever, cough, sore throat, myalgia, and fatigue, and thus represents a persistent, recurring challenge for public health [7]. More importantly, prevention and treatment currently rely on annual vaccination, antiviral drugs such as the neuraminidase inhibitors Oseltamivir, zanamivir, and peramivir, as well as M2 ion channel blockers like amantadine or novel “cap snatching” polymerase inhibitors, in addition to supportive symptomatic care [6,8]. However, antiviral resistance, fluctuating vaccine effectiveness, and limited global access underscore the ongoing need for improved vaccines, novel, effective antiviral agents, and equitable healthcare strategies [9]. Taken together, preparedness for the emergence of highly pathogenic IAV strains with pandemic potential, particularly those of H_1_N_1_ background, remains essential.

Respiratory syncytial virus (RSV) is another major cause of acute respiratory disease and contributes substantially to the global burden of lower respiratory tract infections, particularly in infants and older adults [10,11]. The World Health Organization estimates that RSV causes over 3.6 million hospitalizations and about 100,000 deaths annually among children under five. Most pediatric deaths occur in low- and middle-income countries with limited access to supportive medical care [12,13]. Clinical management remains largely supportive, and licensed therapeutic antivirals are limited [14]. Preventive options have expanded in recent years. For instance, long-acting monoclonal antibodies for infants and maternal immunization can protect young infants during their first RSV season. Furthermore, several prefusion F-based vaccines (e.g., Arexvy, Abrysvo, and mResvia) are licensed for older adults [13]. Nevertheless, uneven global access to these interventions highlights a continuing need for safe, readily available antivirals active against RSV [12].

Natural substances have been used for centuries to counteract infections, even though, for most compounds, their precise antiviral modes of action have not been fully elucidated yet [15]. They have been applied both prophylactically and therapeutically to prevent deterioration of virus-induced diseases, particularly in the context of newly emerging or re-emerging viruses [16]. Because many natural products act broadly against multiple viruses, they have substantial potential to strengthen pandemic preparedness [15]. Moreover, a large number of natural antivirals that either target host cell factors and/or directly neutralize virions may reduce the likelihood of resistance development. Finally, owing to their natural origin and long-standing use, many of these compounds are well tolerated, although systematic assessment of safety and efficacy remains indispensable [15,16,17].

European black elderberry (*Sambucus nigra* L.) has long been employed in traditional medicine to alleviate symptoms of respiratory tract infections. Recent in vivo investigations in animals, as well as clinical studies, indicate that black elderberry fruit preparations can shorten disease duration and lessen symptom severity in upper respiratory infections [18,19,20,21]. In addition, the antiviral activity of a black elderberry fruit extract standardized to anthocyanins and phenolic compounds has been demonstrated in vitro against SARS-CoV-2 (including variants) and IAV [22,23]. Furthermore, a crude ethanol extract of *Sambucus nigra* has exhibited antiviral activity against the infectious bronchitis virus, supporting its potential as a countermeasure against diverse respiratory viruses [24].

Carrageenans represent high-molecular-weight, polysulfated polysaccharides, generally obtained from red seaweeds (Rhodophyta), which have been processed worldwide in the pharmaceutical industry, in foods, and in cosmetics. The U.S. Food and Drug Administration generally classifies them as safe (GRAS). Three main forms are employed commercially—iota, kappa, and lambda carrageenan—and they differ in their degree of sulfation, solubility, and gelling properties [25]. In contrast to low-molecular-weight kappa- and lambda-carrageenan, high-molecular-weight iota-carrageenan (MW ≥ 1100 kDa; IC) does not enter cells and is regarded as biologically inert with respect to adverse and immunomodulatory effects [26,27,28,29]. The antiviral action of IC is well established and has been shown in vitro as well as in clinical studies for a variety of respiratory viruses [30,31,32,33,34,35,36]. Antiviral activity has also been reported for lambda-carrageenan against SARS-CoV-2 [37]. As elderberry fruit preparations are derived from an edible berry, and IC represents a food-grade polysaccharide, the current study addresses food-derived, orally applicable ingredients with potential local antiviral activity.

Here, we describe for the first time that a black elderberry fruit extract containing 3.2% anthocyanins (EC), as well as low dosages of IC, efficiently inhibits the replication of RSV in vitro. Furthermore, this is the first-time report that EC as well as IC neutralize virions derived from SARS-CoV-2, IAV, and RSV extracellularly in a dose-dependent manner. Most importantly, we could show that the combinatory treatment of IC with EC exhibits a synergistic antiviral activity against IAV, SARS-CoV-2, and RSV. In addition, we analyzed the activities of IC and EC derived from newly produced and matrix-standardized lozenges in mono- and combinatory treatment, altogether confirming their synergistic antiviral activities. The cumulative data suggest that a combination of IC and EC might be considered as a novel antiviral against SARS-CoV-2, IAV, and RSV, and maybe other respiratory viruses.

## 2. Materials and Methods

### 2.1. Inhibitors

Liquid European black elderberry (*Sambucus nigra* L.) fruit extract, branded as ElderCraft^®^ and designated EC, was provided by Iprona Lana SpA (Lana, Italy). EC is a standardized aqueous extract containing at least 3.2% total anthocyanins, with anthocyanin content serving as the primary specification marker. Detailed phytochemical characterization and anthocyanin stability of this extract were reported previously [22,23]. The extract used in this study is identical to the EC used in our previously published study. Thus, previous information about the phytochemical characterization and anthocyanin stability is also relevant in this study. In addition, all experiments were performed with the same EC batch.

**Figure 1:**
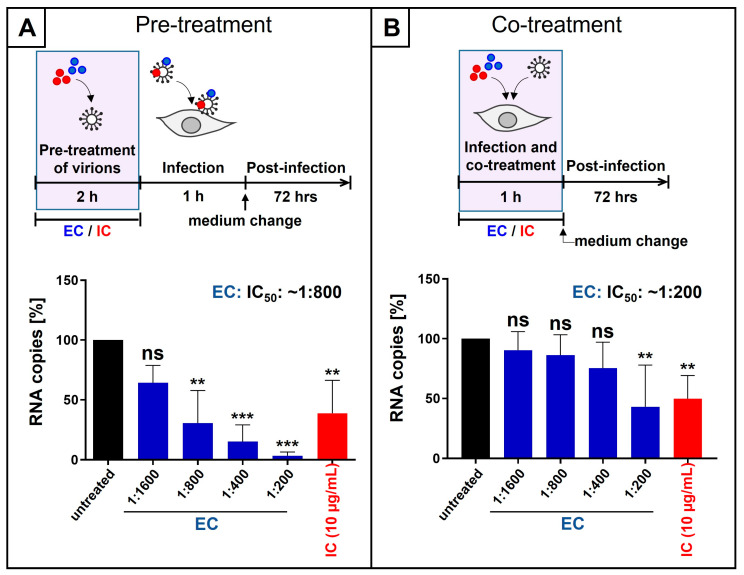
Liquid European black elderberry extract (EC) and iota-carrageenan (IC) inhibit the replication of SARS-CoV-2 Omicron in Calu-3 cells when virions were preincubated before infection (**A**) or when inhibitors were added during infection (**B**). (**A**) SARS-CoV-2 Omicron virus stocks were preincubated with indicated dilution steps of EC or 10 µg/mL IC for two hours, and Calu-3 cells were infected at an MOI of 2 × 10^−2^ for 1 h. After infection and removal of the input virus, medium without interventions was added. Cell culture supernatants were harvested 3 days post-infection. The virions were analyzed by qRT-PCR. Bars show mean values of three independent experiments ± standard deviation. Statistical analysis was performed using a multiple comparison Kruskal–Wallis test (ANOVA) followed by Dunn’s post hoc test (** *p* < 0.0052, *** *p* < 0.0004, and ns = not significant versus the untreated control). (**B**) Calu-3 cells were infected with SARS-CoV-2 Omicron at an MOI of 2 × 10^−2^ for 1 h. During the infection, the indicated dilution steps of EC or 10 µg/mL IC were added. After infection and removal of the input virus as well as EC or IC, medium without interventions was added. Cell culture supernatants were harvested 3 days post-infection. The virions were analyzed by qRT-PCR. Bars show mean values of five independent experiments ± standard deviation. Statistical analysis was performed using a multiple comparison Kruskal–Wallis test (ANOVA) followed by Dunn’s post hoc test (** *p* < 0.0037 and ns = not significant versus the untreated control).

**Figure 2:**
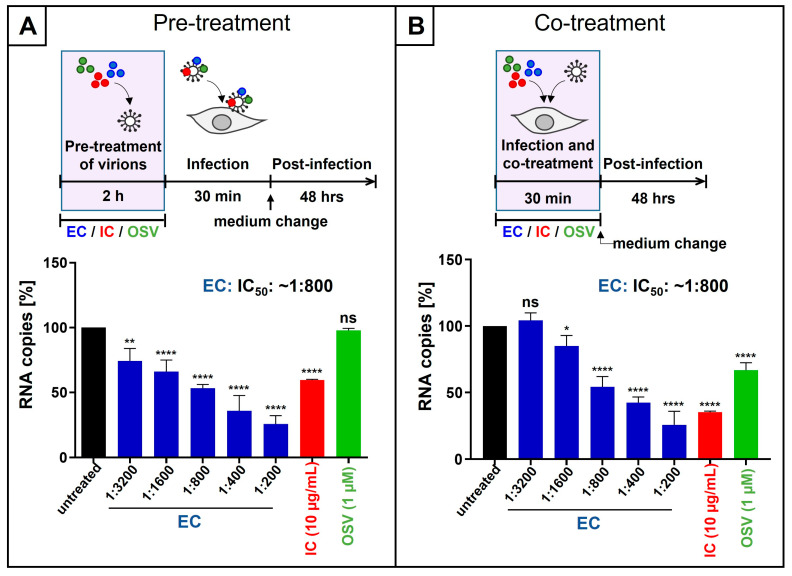
EC and IC inhibit the replication of IAV_PR8_ in MDCKII cells when virions were preincubated before infection (**A**) or when inhibitors were added during infection (**B**). (**A**) IAV_PR8_ virus stocks were preincubated with indicated dilution steps of EC, 10 µg/mL IC, or 1 µM Oseltamivir (OSV) phosphate for two hours. Then, MDCKII cells were infected with the preincubated virions at an MOI of 0.01 for 30 min. After infection and removal of the input virus, medium without interventions was added. Cell culture supernatants were harvested 2 days post-infection. The virions were analyzed by qRT-PCR. Bars show mean values of three independent experiments ± standard deviation. Statistical analysis was performed using a multiple comparison Kruskal–Wallis test (ANOVA) followed by Dunn’s post hoc test (** *p* = 0.0016, **** *p* < 0.0001, and ns = not significant versus the untreated control). (**B**) MDCKII cells were infected with IAV_PR8_ at an MOI of 0.01 for 30 min. During the infection period, the indicated dilution steps of EC, 10 µg/mL IC, or 1 µM OSV phosphate were added. After infection and removal of the input virus as well as EC, IC, and Oseltamivir, medium without interventions was added. Cell culture supernatants were harvested 2 days post-infection. The virions were analyzed by qRT-PCR. Bars show mean values of three independent experiments ± standard deviation. Statistical analysis was performed using a multiple comparison Kruskal–Wallis test (ANOVA) followed by Dunn’s post hoc test (* *p* = 0.0473, **** *p* < 0.0001, and ns = not significant versus the untreated control).

**Figure 3:**
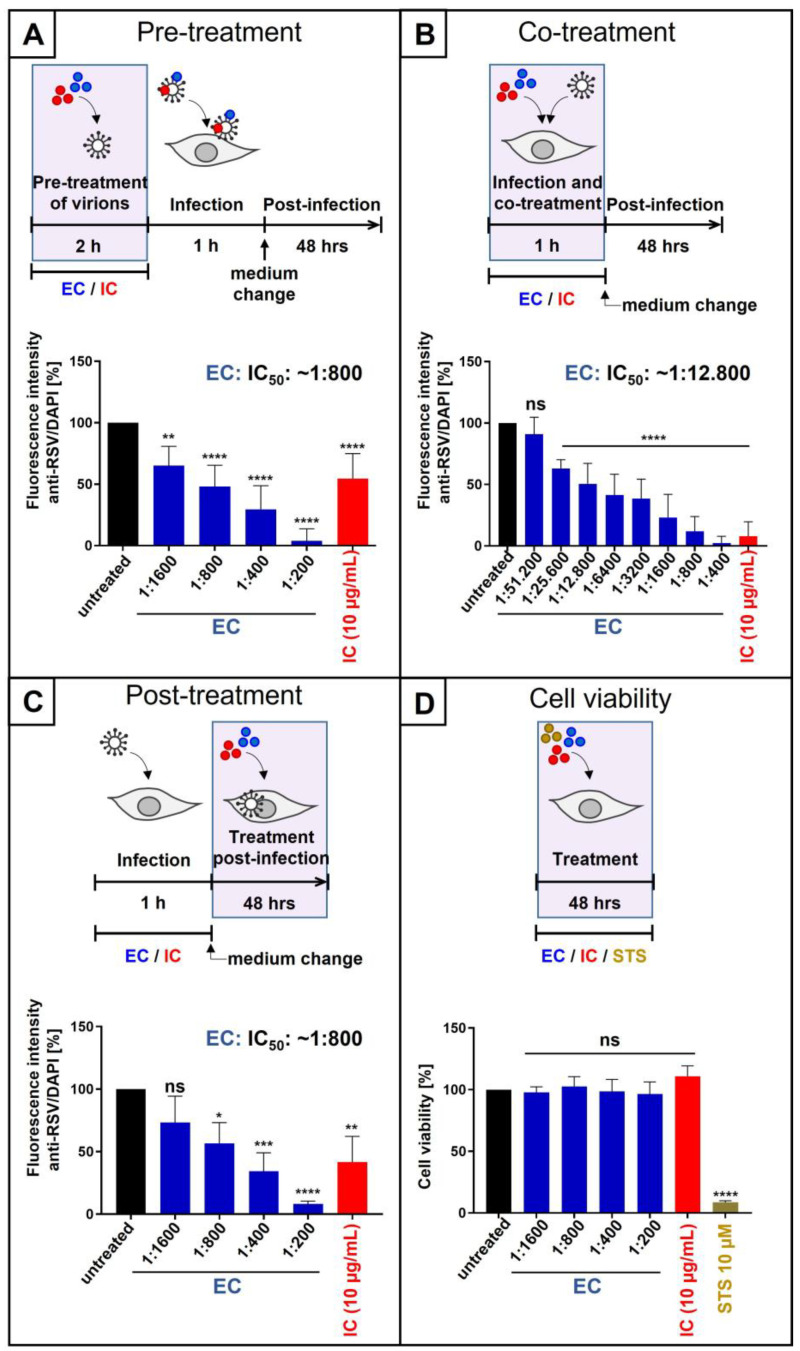
EC and IC inhibit the replication of RSV in HEp-2 cells when virions were preincubated before infection (**A**), when added during infection (**B**), or when added post-infection (**C**), without influencing cell viability (**D**). (**A**) RSV (A2 strain) virus stocks were preincubated with indicated dilution steps of EC or 10 µg/mL IC for two hours. Then, HEp-2 cells were infected with the preincubated virions at an MOI of 0.1 for 1 h. Two days post-infection, cells were fixed with 1% PFA, permeabilized with 0.1% Triton X, and blocked with 1% BSA. Following staining with a goat anti-RSV polyclonal antibody overnight and staining with a donkey anti-goat IgG H&L AlexaFluor 488 antibody for 45 min, as well as DAPI for 10 min, the fluorescence output of AlexaFluor 488 and DAPI was quantitatively analyzed. Bars show mean values of six independent experiments ± standard deviation. Statistical analysis was performed using a multiple comparison Kruskal–Wallis test (ANOVA) followed by Dunn’s post hoc test (** *p* = 0.0021 and **** *p* < 0.0001 versus the untreated control). (**B**) HEp-2 cells were infected with RSV (A2 strain) at an MOI of 0.1 for 1 h. During the infection, the indicated dilution steps of EC or 10 µg/mL IC were added. After infection and removal of the input virus as well as EC or IC, medium without interventions was added. Two days post-infection, antibody staining was performed as described in (**A**). Bars show mean values of at least four independent experiments ± standard deviation. Statistical analysis was performed using a multiple comparison Kruskal–Wallis test (ANOVA) followed by Dunn’s post hoc test (**** *p* < 0.0001 and ns = not significant versus the untreated control). (**C**) HEp-2 cells were infected with RSV (A2 strain) at an MOI of 0.1. One hour after infection and removal of the input virus, cells were treated with the indicated dilution steps of EC or 10 µg/mL IC. Two days post-infection, antibody staining was performed as described under (**A**). Bars show mean values of three independent experiments ± standard deviation. Statistical analysis was performed using a multiple comparison Kruskal–Wallis test (ANOVA) followed by Dunn’s post hoc test (**** *p* < 0.0001, *** *p* = 0.0008, ** *p* = 0.0021, * *p* = 0.0167, and ns = not significant versus the untreated control). (**D**) Following treatment with indicated dilutions of EC or 10 µg/mL IC for two days, the influence on cell viability in uninfected HEp-2 cells was measured by neutral red uptake assay. Staurosporine (STS, 10 µM) was used as a positive control. Bars represent means of three independent experiments ± SD. Statistical analysis was performed using a multiple comparison Kruskal–Wallis test (ANOVA) followed by Dunn’s post hoc test (**** *p* < 0.0001 and ns = not significant versus the untreated control).

**Figure 4:**
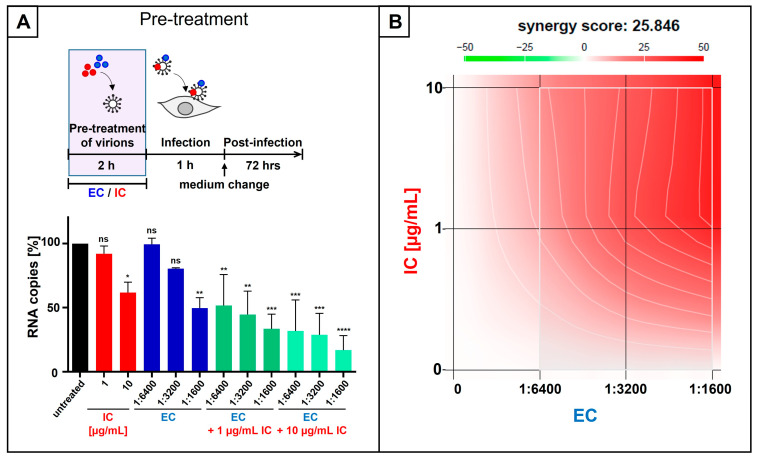
Synergistic antiviral activity of the combinatory treatment of IC and EC following infection with SARS-CoV-2 Omicron. (**A**) SARS-CoV-2 Omicron virus stocks were preincubated with indicated dilution steps of EC, concentrations of IC, or the combination of both (green bars) for two hours. Then, Calu-3 cells were infected with the preincubated virions at an MOI of 2 × 10^−2^ for 1 h. After infection and removal of the input virus, medium without interventions was added. Cell culture supernatants were harvested 3 days post-infection. The virions were analyzed by qRT-PCR. Bars show mean values of three independent experiments ± standard deviation. Statistical analysis was performed using a multiple comparison Kruskal–Wallis test (ANOVA) followed by Dunn’s post hoc test (* *p* < 0.0458, ** *p* < 0.0075, *** *p* < 0.0003, **** *p* < 0.0001, and ns = not significant versus the untreated control). (**B**) Interaction profile of IC and EC for determining the synergy in the inhibition of the replication capacity of SARS-CoV-2 Omicron. Drug interactions were analyzed using the reference model of Bliss independence. The illustration was created using the open-source and free web application SynergyFinder (https://synergyfinder.aittokallio.group/20260409095506952334/ (accessed on 27 January 2026)). The synergy calculations were performed on data derived from the experiments in Calu-3 cells. The data represent the means of 3 independent experiments. A color-coded interaction graphic was used to illustrate the Bliss synergy scores. High synergy scores are colored in red.

**Figure 5:**
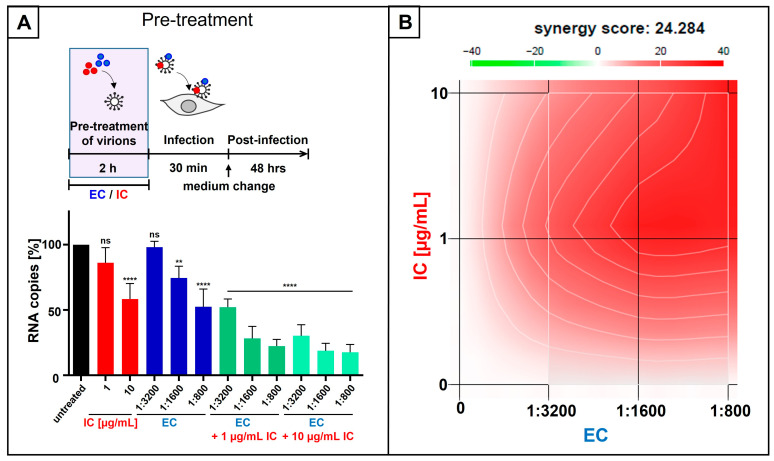
Synergistic antiviral activity of the combinatory treatment of IC and EC following infection with IAV_PR8_. (**A**) IAV_PR8_ virus stocks were preincubated with indicated dilution steps of EC, concentrations of IC, or the combination of both (green bars) for two hours. Then, MDCKII cells were infected with the preincubated virions at an MOI of 0.01 for 30 min. After infection and removal of the input virus, medium without interventions was added. Cell culture supernatants were harvested 2 days post-infection. The virions were analyzed by qRT-PCR. Bars show mean values of three independent experiments ± standard deviation. Statistical analysis was performed using a multiple comparison Kruskal–Wallis test (ANOVA) followed by Dunn’s post hoc test (** *p* = 0.0097, **** *p* < 0.0001, and ns = not significant versus the untreated control). (**B**) Interaction profile of IC and EC for determining the synergy in the inhibition of the replication capacity of IAV_PR8_. Drug interactions were analyzed using the reference model of Bliss independence. The illustration was created using the open-source and free web application SynergyFinder. The synergy calculations were performed on data derived from the experiments in MDCKII cells. The data represent the means of 3 independent experiments. A color-coded interaction graphic was used to illustrate the Bliss synergy scores. High synergy scores are colored in red.

**Figure 6:**
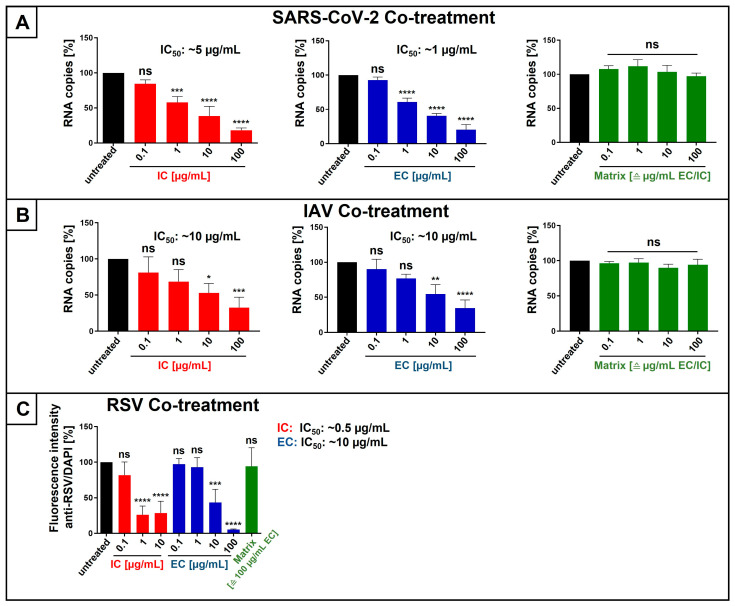
EC and IC, extracted from lozenges, inhibit the replication of SARS-CoV-2 Omicron (**A**), IAV_PR8_ (**B**), and RSV (**C**) when added during infection. (**A**) Calu-3 cells were infected with SARS-CoV-2 Omicron at an MOI of 2 × 10^−2^ for 1 h. During the infection, the indicated concentrations of IC (red) or EC (blue) were added. Matrix (green) was added in the corresponding amount for the indicated concentrations of EC or IC (0.1, 1, 10, and 100 µg/mL). After infection and removal of the input virus as well as IC, EC, or matrix, medium without interventions was added. Cell culture supernatants were harvested 3 days post-infection. The virions were analyzed by qRT-PCR. Bars show mean values of three independent experiments ± standard deviation. Statistical analysis was performed using a multiple comparison Kruskal–Wallis test (ANOVA) followed by Dunn’s post hoc test (*** *p* = 0.0002, **** *p* < 0.0001, and ns = not significant versus the untreated control). (**B**) MDCKII cells were infected with IAV_PR8_ at an MOI of 0.01 for 30 min. During the infection, the indicated concentrations of IC (red) or EC (blue) were added. Matrix (green) was added in the corresponding amount for the indicated concentrations of EC or IC (0.1, 1, 10, and 100 µg/mL). After infection and removal of the input virus as well as IC, EC, and matrix, medium without interventions was added. Cell culture supernatants were harvested 2 days post-infection. The virions were analyzed by qRT-PCR. Bars show mean values of three independent experiments ± standard deviation. Statistical analysis was performed using a multiple comparison Kruskal–Wallis test (ANOVA) followed by Dunn’s post hoc test (* *p* = 0.0105, ** *p* = 0.0012, *** *p* = 0.0009, **** *p* < 0.0001, and ns = not significant versus the untreated control). (**C**) HEp-2 cells were infected with RSV (A2 strain) at an MOI of 0.1 for 1 h. During the infection, the indicated concentrations of IC (red) or EC (blue) were added. Matrix (green) was added in the corresponding amount for the highest concentrations of EC (100 µg/mL). After infection and removal of the input virus as well as EC, IC, or matrix, medium without interventions was added. Two days post-infection, cell antibody staining was performed as described under Figure 3A. Bars show mean values of at least four independent experiments ± standard deviation. Statistical analysis was performed using a multiple comparison Kruskal–Wallis test (ANOVA) followed by Dunn’s post hoc test (*** *p* = 0.0001, **** *p* < 0.0001, and ns = not significant versus the untreated control).

**Figure 7:**
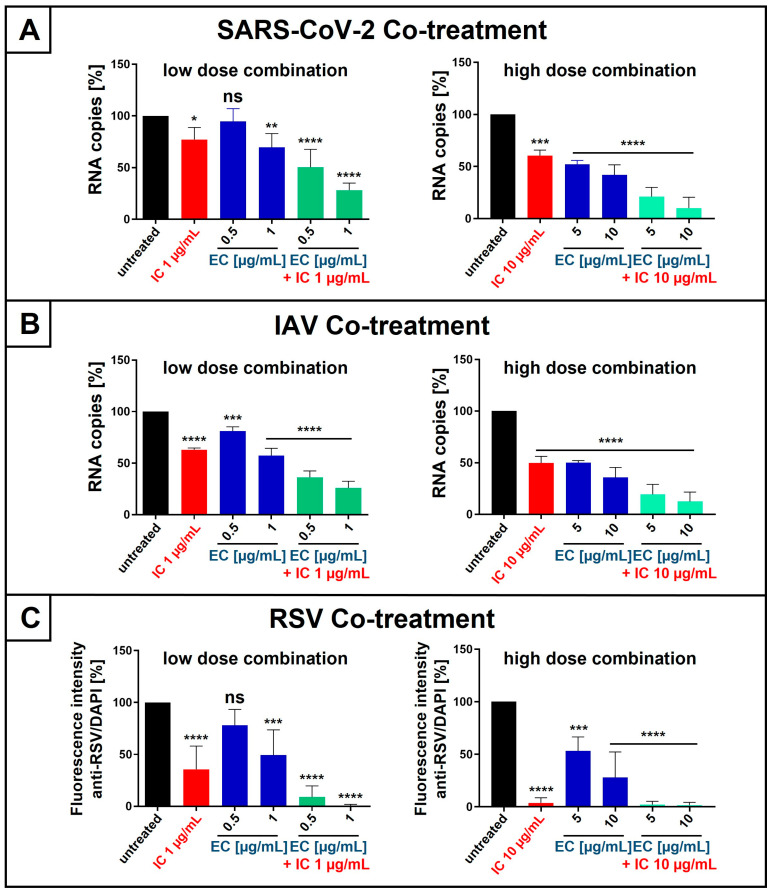
Synergistic antiviral activity of the combinatory treatment of IC and EC, extracted from lozenges, following infection with SARS-CoV-2 Omicron (**A**), IAV_PR8_ (**B**), and RSV (**C**) when added during infection. (**A**) Calu-3 cells were infected with SARS-CoV-2 Omicron at an MOI of 2 × 10^−2^ for one hour. During the infection, cells were treated with indicated low (left side) or high (right side) concentrations of EC or IC or the combination of both. After infection and removal of the input virus, medium without interventions was added. Cell culture supernatants were harvested 3 days post-infection. The virions were analyzed by qRT-PCR. Bars show mean values of four (left side) or three (right side) independent experiments ± standard deviation. Statistical analysis was performed using a multiple comparison Kruskal–Wallis test (ANOVA) followed by Dunn’s post hoc test (* *p* = 0.0489, ** *p* = 0.0073, *** *p* = 0.0001, **** *p* < 0.0001, and ns = not significant versus the untreated control). (**B**) MDCKII cells were infected with IAV_PR8_ at an MOI of 0.01 for 30 min. During the infection, cells were treated with indicated low (left side) or high (right side) concentrations of EC or IC or the combination of both. After infection and removal of the input virus, medium without interventions was added. Cell culture supernatants were harvested 2 days post-infection. The virions were analyzed by qRT-PCR. Bars show mean values of four (left side) or three (right side) independent experiments ± standard deviation. Statistical analysis was performed using a multiple comparison Kruskal–Wallis test (ANOVA) followed by Dunn’s post hoc test *** *p* = 0.0002 and **** *p* < 0.0001 versus the untreated control). (**C**) HEp-2 cells were infected with RSV (A2 strain) at an MOI of 0.1 for one hour. During the infection, cells were treated with indicated low (left side) or high (right side) concentrations of EC or IC or the combination of both. After infection and removal of the input virus, medium without interventions was added. Two days post-infection, cell antibody staining was performed as described under Figure 3A. Bars show mean values of five (left side) or four (right side) independent experiments ± standard deviation. Statistical analysis was performed using a multiple comparison Kruskal–Wallis test (ANOVA) followed by Dunn’s post hoc test (*** *p* = 0.0001, **** *p* < 0.0001, and ns = not significant versus the untreated control).

**Figure 8:**
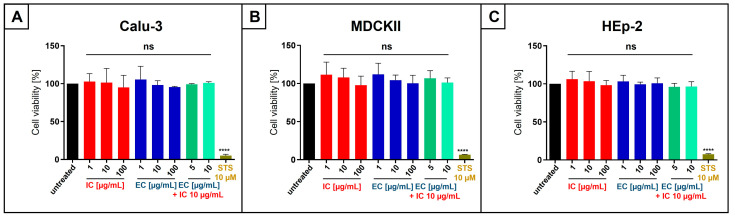
EC and IC, extracted from lozenges as mono- or combinatory treatment, have no influence on the viability of Calu-3 (**A**), MDCKII (**B**), or HEp-2 cells (**C**). (**A**–**C**) Following treatment with indicated concentrations of IC, EC, or the combination of both for three (**A**) or two (**B**,**C**) days, the influence on cell viability in uninfected cells was measured by neutral red uptake assay. Staurosporine (STS, 10 µM) was used as a positive control. Bars represent means of three independent experiments ±SD. Statistical analysis was performed using a multiple comparison Kruskal–Wallis test (ANOVA) followed by Dunn’s post hoc test (**** *p* < 0.0001 and ns = not significant versus the untreated control).

In experiments using the liquid extract (Figure 1, Figure 2, Figure 3, Figure 4 and Figure 5), EC concentrations are reported as dilution factors of the supplied inhibitor stock. In contrast, experiments with lozenge-derived EC (Figure 5, Figure 6, Figure 7 and Figure 8) working concentrations are reported in µg/mL. For the reason of the specific lozenge production process conditions, only powdered black elderberry extracts had to be mixed with the dry matrix, finally enabling concentration measurements of EC in µg/mL.

Iota-(Gelcarin PH 379)-carrageenan was purchased from DuPont (formerly FMC Biopolymers) (both Philadelphia, PA, USA). The dry polymer powders were dissolved in cell culture water (B. Braun Melsungen AG, Melsungen, Germany) to a final concentration of 1.2 mg/mL containing 0.5% NaCl (Merck KGaA, Darmstadt, Germany). This stock solution was sterile-filtered through a 0.22 µm filter (Sarstedt, Nuembrecht, Germany) and stored at 4 °C until use.

Oseltamivir phosphate was purchased from Sigma-Aldrich (St. Louis, MO, USA), dissolved in water, and stored at −20 °C until use.

### 2.2. Preparation of Lozenges

For the preparation of lozenges, isomalt was combined with deionized water and heated under gentle agitation until fully liquefied, then further heated to 150–153 °C with continuous agitation. The molten mixture was subsequently cooled to 140 °C by immersion in a room-temperature water bath while maintaining gentle agitation. At 140 °C, IC/EC and various combinations were added by evenly distributing the dry components across the surface of the melt and allowing them to dissolve under constant mixing. The mixture was manually stirred for 30 s to ensure uniform dispersion, poured into pre-prepared molds, and allowed to cool at room temperature until fully solidified. The following mono- and combi-lozenges were produced under aseptic conditions (mg per lozenge): 10 mg IC, 5 mg EC, 10 mg EC, 10 mg IC + 5 mg EC, 10 mg IC + 10 mg EC, and, as a control, isomalt without IC or EC, designated as matrix. One lozenge contains an average of 3.3 g of isomalt. For infection experiments, three lozenges were dissolved in 30 mL PBS for each IC or EC intervention in order to equalize inhibitor concentrations. The following concentrations were prepared: 1 mg/mL IC, 0.5 mg/mL EC, 1 mg/mL EC and matrix (containing 0.33 g/mL isomalt), 1 mg/mL IC + 0.5 mg/mL EC, and 1 mg/mL IC + 1 mg/mL EC. All solutions were stored at 4 °C until use. For low-dose combinations, the following concentrations were used for the conducted experiments: 1 mg/mL IC + 0.5 mg/mL EC, 1 mg/mL IC + 1 mg/mL EC; for high-dose combinations: 10 mg/mL IC + 5 mg/mL EC, 10 mg/mL IC + 10 mg/mL EC.

### 2.3. Viruses

The clinical SARS-CoV-2 Omicron variant was isolated from an anonymized residual swab sample of a patient infected with the SARS-CoV-2 Omicron variant, as described previously [38]. For the generation of new virus stock, virus-containing cell culture supernatant was harvested 72 h post-infection (hpi) and passed through a 0.45 μm pore-size filter. All virus stocks were stored at −80 °C until further usage. For titer determination of SARS-CoV-2 virus stocks, Calu-3 cells were infected with serial dilutions of the virus stock over 72 h. Afterwards, cells were fixed (4% PFA), permeabilized (0.5% Triton/PBS), blocked (1% BSA/PBS-T), and finally stained with a SARS-CoV-2 NP antibody (Biozol, Eching, Germany). The endpoint of virus infection was analyzed via fluorescence microscopy, and viral titer was calculated by the method of Reed and Muench [39].

The influenza A virus isolate (IAV) A/Puerto Rico/8/34 (PR8) was provided by Prof. Dr. Matthias Tenbusch (Harald zur Hausen Institute of Virology, Erlangen, Germany). For the generation of new virus stock, virus-containing cell culture supernatant was harvested 48 hpi and passed through a 0.45 μm pore-size filter. All virus stocks were stored at −80 °C until further usage. Viral titers were determined by a plaque assay. For titer determination of IAV_PR8_ virus stocks, MDCKII cells were infected with serial dilutions of the virus stock for 3 h. Afterwards, cells were overlaid with 1% purified agar (Oxoid, Wesel, Germany) and incubated at 37 °C for 4 days. The endpoint of virus infection was analyzed by counting plaque-forming units (PFU) visualized following incubation of the cells with MTT 3-(4,5-Dimethylthiazol-2-yl)-2,5-diphenyltetrazolium bromide for 3 h. Viral titer was calculated by the method of Reed and Muench [39].

The RSV isolate (RSV A2 strain) was provided by Prof. Dr. Matthias Tenbusch (Harald zur Hausen Institute of Virology, Erlangen, Germany). For the generation of new virus stock, HEp-2 cells were infected with RSV, followed by a second round of infection using the first infection supernatant. Virus-containing cell culture supernatant was harvested 72 h later and passed through a 0.45 μm pore-size filter. Following ultracentrifugation through a 20% sucrose cushion at 4 °C for 3 h at 10,600 rpm, all virus stocks were stored at −80 °C until further usage. Viral titers were determined by an endpoint titration assay.

### 2.4. Infection Experiments

Calu-3 cells were inoculated with SARS-CoV-2 Omicron with a multiplicity of infection (MOI) of 2 × 10^−2^ and co-treated with interventions for 1 h, followed by medium change without further treatment. For pre-treatment conditions, 10 µL of a SARS-CoV-2 Omicron virus stock was pre-incubated with the indicated intervention(s) for 2 h at 37 °C in Minimal Essential Medium (MEM). Then, 10 µL of the pre-incubated virus stock was transferred into 1.5 mL of MEM without FCS. Thereby, the activity of the inhibitor(s) was diluted by 2.18 log steps, resulting in negligible residual amounts. Subsequently, 250 µL of medium, containing the pretreated virions, was used for infection of Calu-3 cells for 1 h, with an MOI of ~2 × 10^−2^ (based on the specific infectivity calculated for the virus stock before pre-treatment with the interventions). Following infection, the medium was removed, and cells were washed twice with PBS. Then, a new MEM without inhibitors was added. At 72 hpi, virus-containing cell culture supernatants were harvested and incubated for 10 min at 95 °C, and finally used for qRT-PCR analysis.

MDCKII (Madin-Darby Canine Kidney II) cells were inoculated with IAV_PR8_ (MOI: 0.01) and co-treated with interventions for 30 min, followed by medium change without further treatment. Alternatively, 1 µL from an IAV_PR8_ stock was pre-incubated with the indicated intervention(s) in 100 µL of Dulbecco’s Modified Eagle Medium (DMEM) for 2 h at 37 °C. Then, 2 µL of the pre-incubated virus stock was transferred into 150 µL of PBS with 1% bovine serum albumin (BSA), 100 U/mL penicillin, 100 μg/mL streptomycin, and 0.01% CaMg. Subsequently, 150 µL of this medium, containing the pretreated virions, was used for infection of MDCKII cells for 30 min with an MOI of ~0.01 (based on the specific infectivity calculated for the virus stock before pre-treatment with the interventions). Thereby, the activity of the inhibitors was diluted by 1.88 log steps, resulting in negligible residual amounts. Following the removal of the medium and two PBS washing steps, new DMEM without interventions was added. At 48 hpi, virus-containing cell culture supernatants were harvested and incubated for 10 min at 95 °C, and finally used for qRT-PCR analysis.

HEp-2 cells were inoculated with RSV (MOI: 0.1) and either co-treated with interventions for 1 h or treated with interventions following 1 h of infection (post-treatment). Subsequently, the medium was changed. Alternatively, an RSV stock was pre-diluted 1:10 with DMEM. Next, 10 µL of the pre-diluted virus stock was pre-incubated with the indicated intervention(s) for 2 h at 37 °C. Then, 4.15 µL of the pre-incubated virus stock was transferred into 1 mL DMEM without FCS. Subsequently, 50 µL of this medium, containing the pretreated virions, was used for infection of HEp-2 cells for 1 h with an MOI of ~0.1 (based on the specific infectivity calculated for the virus stock before pre-treatment with the interventions). Thereby, the activity of the inhibitors was diluted by 2.39 log steps, resulting in negligible residual amounts. Following the removal of the medium and two PBS washing steps, new DMEM without interventions was added. At 48 hpi, immunostaining analysis was conducted.

### 2.5. Immunostaining RSV

For immunostaining, HEp-2 cells were fixed with 1% PFA for 1 h at room temperature. After washing, cells were permeabilized for 10 min with 0.1% Triton and afterwards blocked with 1% BSA for 1 h at 4 °C. Cells were stained with a goat α-RSV polyclonal antibody (Merck, Darmstadt, Germany) (1:1000) for 24 h at 4 °C. Plates were then incubated for 1 h with a donkey anti-goat IgG H&L antibody (AlexaFluor^®^488) (Abcam, Cambridge, UK) for 45 min at room temperature and finally stained with DAPI (Sigma-Aldrich, St. Louis, MO, USA) for 10 min. Finally, the fluorescence output of AlexaFluor488 was quantitatively analyzed with a PerkinElmer VictorX4 (PerkinElmer GmbH, Rodgau, Germany) (at 488 nm) and the fluorescence output of DAPI at 461 nm.

### 2.6. Cell Culture

Calu-3 cells were maintained in Minimal Essential Medium (MEM) containing 10% (*v*/*v*) inactivated FCS, 1 mM L-glutamine, 100 U/mL penicillin, 100 μg/mL streptomycin, 1% MEM NEAA (100×) non-essential amino acid solution, and 1 mM sod ium pyruvate.

MDCKII cells were maintained in Dulbecco’s Modified Eagle Medium (DMEM) containing 10% (*v*/*v*) inactivated FCS, 1 mM L-glutamine, 100 U/mL penicillin, and 100 μg/mL streptomycin.

HEp-2 cells were maintained in DMEM containing 10% (*v*/*v*) inactivated FCS, 1 mM L-glutamine, 100 U/mL penicillin, and 100 μg/mL streptomycin.

### 2.7. Assessment of Cell Viability

To determine the viability of uninfected but treated cells, a neutral red uptake assay was conducted according to a published protocol [40]. Briefly, neutral red (3-amino-7-dimethylamino-2-methyl-phenazine hydrochloride) (Sigma-Aldrich, St. Louis, MO, USA) was added for 2 h to the treated cells at 37 °C. Then, cells were washed with PBS, and the neutral red dye was extracted with an acidified ethanol solution and analyzed with a Perkin Elmer VictorX4 (at 540 nm).

### 2.8. Determination of the Amount of Viral RNA Copies from Released Viruses by qRT-PCR

The amount of viral SARS-CoV-2 RNA copies in the virus-containing samples was quantified by real-time RT-PCR using the Luna Universal Probe One-Step RT-PCR Kit from New England Biolabs (Cat: E3006L, Ipswich, MA, USA). This kit allows reverse transcription, cDNA synthesis, and PCR amplification in a single step. Samples were analyzed by 7500 software v2.3 (Applied Biosystems, Waltham, MA, USA). PCR primers were designed and used as described previously in [41]. Thereby, the polynucleotide sequence contains parts of the SARS-CoV-2 Envelope (E) and RNA-dependent RNA polymerase (RdRp) genes and was used as a standard for the determination of viral RNA copies in the experiments. The sequences of the used primers were RdRp_forward (fwd): 5′-GTG-ARA-TGG-TCA-TGT-GTG-GCG-G-3′ and RdRp_reverse (rev) 5′-CAR-ATG-TTA-AAS-ACA-CTA-TTA-GCA-TA-C-3′. Probe was 5′-CAG-GTG-GAA-/ZEN/CCT-CAT-CAG-GAG-ATG-C -3′ (Label: FAM/IBFQ Iowa Black FQ). A dsDNA-polynucleotide sequence (Integrated DNA Technologies, Coralville, IA, USA) was used as a positive control: 5′-TAA-TAC-GAC-TCA-CTA-TAG-GGT-ATT-GAG-TGA-AAT-GGT-CAT-GTG-TGG-CGG-TTC-ACT-ATA-TGT-TAA-ACC-AGG-TGG-AAC-CTC-ATC-AGG-AGA-TGC-CAC-AAC-TGC-TTA-TGC-TAA-TAG-TGT-TTT-TAA-CAT-TTG-GAA-GAG-ACA-GGT-ACG-TTA-ATA-GTT-AAT-AGC-GTA-CTT-CTT-TTT-CTT-GCT-TTC-GTG-GTA-TTC-TTG-CTA-GTT-ACA-CTA-GCC-ATC-CTT-ACT-GCG-CTT-CGA-TTG-TGT-GCG-TAC-TGC-TGC-AAT-ATT-GTT-3′. Generating a series of dilutions (10^4^, 10^5^, 10^6^, and 10^7^ copies/mL) of this standard, the experiments were quantified using a standard curve to obtain absolute values of RNA copies in the sample.

The amount of viral IAV RNA copies in the virus-containing samples was quantified by real-time PCR GoTaq^®^ Probe qPCR one-step Kit from Promega (Cat: A6120, Madison, WI, USA). This kit allows reverse transcription, cDNA synthesis, and PCR amplification in a single step. Samples were analyzed by 7500 software v2.3 (Applied Biosystems, Waltham, MA, USA). PCR primers (Integrated DNA Technologies, Coralville, IA, USA) contain parts of the IAV matrix gene. The sequences of the used primers were: 5′Inf-A-M: 5′-AGA TGA GTC TTC TAA CCG AGG TCG-3′, 3′Inf-A-M: 5′-TGC AAA AAC ATC TTC AAG TCT CTG-3′, and 3′Inf-A-SW-M: 5′-TGC AAA GAC ATC TTC CAG TCT CTG-3′.

### 2.9. Software and Statistics

GraphPad Prism 9.0 was used for statistical analyses and to generate graphs. 7500 software v2.3 was used to evaluate the results obtained by qRT-PCR. To determine the combinatorial effects of the treatment with EC and IC, the open-source and free web application SynergyFinder was used [42]. This program enables the visualization of data on drug combinations by analyzing the drug interactions with the commonly used Bliss independence model [43]. Thereby, a potential drug combination effect is presented as a synergy landscape map over the dose matrix. For synergy analysis, mean inhibition values from three independent experiments were entered into SynergyFinder and evaluated using the Bliss independence model. Thereby, Bliss scores > 10 indicate synergistic activity, scores between −10 and 10 additive, and scores < −10 antagonistic activity. Statistical significance of the underlying experimental groups was assessed using a Kruskal–Wallis multiple-comparison test followed by Dunn’s post hoc test. Formal Bliss scoring was applied to the matrix-based combination experiments shown in Figure 4 and Figure 5.

## 3. Results

### 3.1. European Black Elderberry Extract (EC) and Iota-Carrageenan (IC) Inhibit the Replication of SARS-CoV-2 in Calu-3 Cells Under Pre- and Co-Treatment Conditions

In our previous reports, we observed strong antiviral activities against SARS-CoV-2 and IAV when EC or IC were added to cell culture media after infection [22,23,32]. This treatment, termed post-infection, always started when the input virus was removed from the cell culture. Therefore, no information exists about potential virus-neutralizing activities, e.g., in situations when IC or EC would be present prior to and/or during the infection process, conditions that would be more practice-oriented, namely to protect in real virus exposure situations.

In order to investigate potential virus-neutralization activities of EC and IC on extracellular virions, we established a novel protocol, described in detail in the Materials and Methods Section. Since we failed to purify virions after treatment with inhibitors, we successfully treated highly concentrated virus stocks with several inhibitor concentrations followed by several log-dilution steps, resulting in negligible residual amounts of EC or IC in those virus input solutions, which we termed pretreated.

To analyze the influence of extracellular pre-treatment of virions derived from SARS-CoV-2 Omicron on virus replication, virus stocks were pretreated with either dilutions of EC or 10 µg/mL IC (Figure 1A). Following infection of Calu-3 cells [44] for 1 h, the inoculum was removed, and fresh medium without EC and/or IC was added. At three days post-infection (dpi), cell culture supernatants were collected, and virus production was assessed by quantitative RT-PCR (qRT-PCR) (Figure 1A). Pre-treatment of SARS-CoV-2 virions with EC led to a dose-dependent inhibition of viral replication, resulting in an IC_50_ of ~1:800 dilution of EC and complete block of the production of progeny virions at a dilution of 1:200 EC. Treatment with 10 µg/mL IC led to a ~50% reduction (Figure 1A). Thereby, the log-dilution steps to minimize inhibitor carryover resulted in a residual concentration of 1:121,000-fold diluted EC and 66 ng/mL IC, respectively.

To investigate the antiviral effect under co-treatment conditions, EC or IC was added during the infection of Calu-3 cells with SARS-CoV-2 Omicron for 1 h. Following medium change, cells were cultivated in the absence of the inhibitors. Three dpi, cell culture supernatants were harvested, and qRT-PCR was performed to analyze virus production (Figure 1B). Co-treatment of SARS-CoV-2 virions and Calu-3 cells during infection caused a reduction in virus replication by ~50% for a dilution of 1:200 EC and by ~50% for 10 µg/mL IC (Figure 1B).

We previously published that EC, derived from liquid black elderberry, had no influence on cell viability of Calu-3 cells up to a dilution of 1:100, which points towards a broad therapeutic window [22].

### 3.2. European Black Elderberry Extract and Iota-Carrageenan Inhibit the Replication of IAV in MDCKII Cells Under Pre- and Co-Treatment Conditions

Next, we analyzed whether extracellular pre-treatment of virions derived from IAV_PR8_ with EC or IC also blocked virus replication. Therefore, virus stocks were incubated for 2 h with either different dilutions of EC or 10 µg/mL IC (Figure 2A). MDCKII cells [45] were infected for 30 min, the inoculum was removed, and fresh medium without EC and/or IC was added. Two dpi, cell culture supernatants were harvested, and qRT-PCR was performed to analyze virus production (Figure 2A). Thereby, pre-treatment of IAV virions with EC resulted in a dose-dependent reduction in viral replication, with an IC_50_ of ~1:800 dilution of EC and a block of the production of progeny virions by 80% at a dilution of 1:200 EC. Treatment with 10 µg/mL of IC led to a reduction of ~50% virus replication (Figure 2A). In contrast, pre-treatment of virions with 1 µM Oseltamivir [46] had no influence on IAV replication. Like other neuraminidase inhibitors, Oseltamivir does not act on cell-free virions but rather blocks the release of IAV from producer cells [46]. This negative control furthermore validates our dilution protocol for pre-treatment of viruses, inasmuch as no residual Oseltamivir affected virus replication during the 2-day incubation. Moreover, the log-dilution steps to minimize inhibitor carryover resulted in a residual concentration of 1:60,686-fold diluted EC and 133 ng/mL IC, respectively.

As for SARS-CoV-2, we also investigated the influence of co-treatment with EC or IC during the infection of MDCKII cells with IAV_PR8_ for 30 min. Following infection, the medium was exchanged, and cells were cultivated in new medium without EC or IC. Two dpi, cell culture supernatants were harvested, and qRT-PCR was performed to analyze virus production (Figure 2B). Co-treatment of IAV virions and MDCKII cells during infection resulted in a dose-dependent reduction in virus replication with an IC_50_ of 1:800 dilution for EC and ~7 µg/mL for IC (Figure 2B). Treatment with 1 µM Oseltamivir resulted in a moderate block of IAV replication by ~30%. In contrast to the pre-treatment setting (Figure 2A), under co-treatment, this inhibitor was present in cell culture during the 30 min infection period and could deploy its inhibitory effect on cells.

We previously published that EC, derived from liquid black elderberry, had no influence on cell viability of MDCKII cells up to a dilution of 1:100, which again points towards a broad therapeutic window [23].

### 3.3. European Black Elderberry Extract and Iota-Carrageenan Inhibit the Replication of RSV in HEp-2 Cells

Several clinical trials, as well as animal studies, provided evidence for a therapeutic effect of EC and IC following infection with respiratory viruses in general [18,19,20,21,30,31,32,33,47,48]. However, it has never been experimentally established whether these natural substances exhibit a specific antiviral activity against RSV, one of the major causes of common cold–like illness and lower respiratory tract infections.

Thus, we intended to investigate whether a potential antiviral activity of EC and IC occurs prior to, during, or after infection with RSV. Therefore, we infected HEp-2 cells, a standard cell model for the infection with RSV [49], with an A2 strain of RSV. To analyze the influence of IC and EC on the replication of RSV, we chose the standard readout system of intracellular immunostaining [50,51,52], using an anti-RSV antibody specific for the major RSV antigens.

We first analyzed whether extracellular pre-treatment of RSV stocks had any influence on virus replication. Therefore, virus stocks were pretreated for 2 h with either different dilutions of EC or 10 µg/mL IC, followed by infection of HEp-2 cells for 1 h. After medium change, cultivation was continued in the absence of inhibitors. Two dpi, intracellular immunostaining with an anti-RSV antibody was performed, and the fluorescence intensity was measured (Figure 3A). Thereby, pre-treatment of RSV virions with EC resulted in a dose-dependent inhibition of viral replication, with an IC_50_ of ~1:800 dilution of EC and complete block of replication at a dilution of 1:200 EC. Treatment with 10 µg/mL IC led to ~50% reduction in virus replication (Figure 3A). Thereby, the log-dilution steps to minimize inhibitor carryover resulted in a residual concentration of 1:196,377-fold EC and 40 ng/mL IC, respectively.

We next wanted to investigate whether co-treatment with EC or IC during the infection of HEp-2 cells with RSV has any influence on virus replication. As before for SARS-CoV-2 and IAV (Figure 1B and Figure 2B), after 1 h of infection, the medium was exchanged, and fresh medium without EC or IC was added. Two dpi, intracellular immunostaining was performed (Figure 3B). Co-treatment during infection resulted in a dose-dependent reduction in virus replication with an IC_50_ of ~1:12.800 dilution for EC. Co-treatment with 10 µg/mL IC blocked the replication of RSV by 90% (Figure 3B).

In a third time-of-addition experimental setup, we wanted to analyze whether post-infection treatment with EC or IC can also block the replication of RSV. Therefore, HEp-2 cells were infected with RSV for 1 h, followed by the addition of either different dilutions of EC or 10 µg/mL IC during the 2-day incubation period, revealing that also post-infection treatment causes a dose-dependent reduction in RSV replication with an IC_50_ of ~1:800 dilution of EC and ~50% reduction for 10 µg/mL IC (Figure 3C).

To exclude that EC or IC have nonspecific effects on cell viability, neutral red assays were conducted in uninfected HEp-2 cells under the same conditions as performed during the experimental setup for the post-infection treatment. Thereby, neither EC, up to a dilution of 1:200, nor 10 µg/mL IC had any influence on cell viability while completely blocking RSV replication (Figure 3D). Staurosporine (10 µM) served as a positive control.

### 3.4. Combinatory Treatment with Black Elderberry Fruit Extract and Iota-Carrageenan Exhibits Synergistic Antiviral Activity Against SARS-CoV-2 and IAV

We were able to show that EC and IC neutralize virions (Figure 1A, Figure 2A and Figure 3A). Moreover, it has been well established that the antiviral activity of IC occurs exclusively extracellularly, as it does not enter cells [53]. Thus, it was intriguing to assess a potential combinatory effect of both IC and EC. Particularly, as it was demonstrated previously that EC in combination with the natural compound quinine acts synergistically against SARS-CoV-2 and IAV [23]. Thus, it was legitimate to assess whether co-treatment with EC and IC also results in an additive or even synergistic inhibition of SARS-CoV-2 and IAV.

To examine this issue, SARS-CoV-2 Omicron stocks were pre-incubated for 2 h with serial dilutions of EC, defined concentrations of IC (1 and 10 µg/mL), or both agents in combination (Figure 4A). Calu-3 cells were infected with the pretreated virions for 1 h. Subsequently, the inoculum was removed, and fresh medium without EC and/or IC was added. Cell culture supernatants were collected 3 dpi, and virus production was quantified by qRT-PCR (Figure 4A).

When IC was applied at 10 µg/mL, which corresponds to ~ 50% inhibition of SARS-CoV-2 replication (Figure 1A), the addition of EC (1:6400–1:1600) further decreased viral replication in a dose-dependent manner. This combinatory treatment resulted in up to a 70–85% reduction in replication capacity (Figure 4A). Most importantly, combining the same EC dilution series with only 1 µg/mL IC still produced a pronounced antiviral activity, causing up to 50–70% reduction in replication capacity (Figure 4A). EC (1:6400 or 1:3200) and IC alone (1 µg/mL) did not significantly affect SARS-CoV-2 replication (Figure 4A), while combining those sub-effective doses (1:6400 EC and 1 µg/mL IC) reduced viral replication by 50%, which points towards a strong synergistic antiviral effect.

To quantify the combination effects of EC and IC, we calculated synergy scores using the Bliss independence model, where Bliss scores > 10 indicate synergy, values between −10 and 10 suggest additivity, and scores < −10 imply antagonism. Using this approach, the EC/IC combination yielded a Bliss synergy score of 25.8 for inhibition of SARS-CoV-2 Omicron replication (Figure 4B), supporting a high degree of synergistic activity.

We next investigated whether the combinatory treatment with EC and IC also displays synergistic antiviral activity against IAV. To this end, IAV_PR8_ virus stocks were pre-incubated for 2 h with serial dilutions of EC, two distinct concentrations of IC, or both in combination (Figure 5A). MDCKII cells were infected with the pretreated virions for 30 min, after which the inoculum was removed, and medium without EC or IC was added. Supernatants were harvested 2 dpi, and the virus amount was determined by qRT-PCR (Figure 5A).

Treatment with increasing amounts of EC (1:3200–1:800) in the presence of 10 µg/mL IC—a concentration corresponding to ~50% inhibition of IAV replication in monotherapy (Figure 2A)—resulted in a significant dose-dependent decrease in replication capacity, reaching 70–80% inhibition (Figure 5A). Combining the same EC dilution series with 1 µg/mL IC also produced a strong antiviral effect, reducing viral replication by approximately 50–70% across the tested EC range (Figure 5A).

In contrast, treatment with EC or IC alone had only minor effects on IAV replication at the concentrations tested (Figure 5A). In line with this, neither 1:3200 EC nor 1 µg/mL IC significantly altered viral replication when applied as single agents (Figure 5A). However, the combination of 1:3200 EC with 1 µg/mL IC reduced virus replication by 50%, again pointing towards a synergistic antiviral interaction. A strong Bliss synergy score of 24.28 was calculated (Figure 5B).

Taken together, these data demonstrate that EC and IC act synergistically to inhibit the replication of SARS-CoV-2 and IAV. The mutual enhancement of inhibitor activities at comparatively low concentrations suggests that co-administration of these two natural substances may provide substantial antiviral efficacy, even under natural conditions where inhibitors might be present at diluted and thus lower concentrations.

### 3.5. EC and IC Derived from Lozenges Inhibit the Replication of SARS-CoV-2, IAV, and RSV When Added as Mono-Treatment

We showed that IC and EC are able to neutralize virions extracellularly and thereby act synergistically (Figure 1A, Figure 2A, Figure 3A, Figure 4 and Figure 5). Topical application of IC or EC in the nasopharynx by nasal or oral sprays or lozenges is well established [54,55]. In a previous clinical study, we analyzed the antiviral activity of IC-containing saliva (IC was derived from lozenges) and showed that IC concentration in saliva reached the concentration necessary for inhibition of SARS-CoV-2 replication by 60-fold [56]. Now, we have produced for the first time lozenges that contain both IC and/or EC under comparable matrix conditions, as there are currently no lozenges available containing IC or EC under comparable matrices. As lozenges are usually taken for virus exposure prophylaxis, we decided to test the newly produced lozenges in either mono- or combinatory settings under co-treatment conditions, assuming that these treatment options best mimic in vivo conditions.

For the production of mono- and combi-lozenges used in this study, isomalt (termed as matrix) was combined with IC or EC, as described in detail in the Materials and Methods Section. For the previous experiments presented in Figure 1, Figure 2, Figure 3, Figure 4 and Figure 5, we used liquid black elderberry extract and thus could only provide dilutions of EC. However, for the production of the lozenges, powder from black elderberry extracts was used, which enables us to provide concentrations of EC in µg/mL. The mono-lozenges were dissolved in PBS to reach the following final concentrations: 1 mg/mL IC, 0.5 mg/mL EC, and 1 mg/mL EC.

We first analyzed the antiviral activity of mono-lozenges-derived IC and EC by co-treating either HEp-2 or Calu-3 cells during 1 h of infection with RSV or SARS-CoV-2 Omicron, or MDCKII cells during 30 min of infection with IAV_PR8_. Following infection and medium exchange, cells were cultivated in medium without EC or IC. In the case of SARS-CoV-2 and IAV, at three and two dpi, respectively, cell culture supernatants were collected, and virus production was quantified by qRT-PCR (Figure 6A,B). Regarding RSV, intracellular immunostaining was performed 2 dpi (Figure 6C). As a control, isomalt, representing the matrix of the lozenges without IC or EC, was added during infection (Figure 6A-C).

The addition of IC or EC resulted in a dose-dependent reduction in virus replication for SARS-CoV-2, IAV, and RSV (Figure 6A–C). The IC_50_ values for EC were ~1 µg/mL for SARS-CoV-2 (Figure 6A), ~10 µg/mL for IAV (Figure 6B), and approximately 10 µg/mL for RSV (Figure 6C). The addition of IC resulted in the following IC_50_ values: ~5 µg/mL for SARS-CoV-2 (Figure 6A), ~10 µg/mL for IAV (Figure 6B), and ~0.5 µg/mL for RSV (Figure 6C).

In contrast, in all experimental setups, the addition of matrix in the same amount as for IC or EC (0.1–100 µg/mL) had no influence on virus replication (Figure 6A–C). Thus, a nonspecific effect of the isomalt matrix, used for the production of all types of lozenges, could be excluded.

### 3.6. Combinatory Treatment with Black Elderberry Fruit EXTRACT and Iota-Carrageenan Derived from Lozenges Exhibits Synergistic Antiviral Activity Against SARS-CoV-2, IAV, and RSV

Given our observation that EC and IC exhibit antiviral activity against SARS-CoV-2, IAV, and RSV (Figure 1, Figure 2 and Figure 3) and act synergistically (Figure 4 and Figure 5), we next wanted to analyze whether the combinatory treatment with EC and IC derived from the very same combi-lozenge also exhibits synergistic activity. We therefore used combi-lozenges with IC and EC present in either high (highdose combination; 10 µg/mL IC + 5 µg/mL EC or 10 µg/mL IC + 10 µg/mL EC), or low (lowdose combination; 1 µg/mL IC + 0.5 µg/mL EC or 1 µg/mL IC + 1 µg/mL EC) concentrations. As a control, mono-lozenges with single concentrations of IC or EC were used: 1 or 10 µg/mL IC and 0.5, 1, 5, or 10 µg/mL EC.

As for the monotherapy (Figure 6), either HEp-2 or Calu-3 cells were co-treated with defined concentrations of IC or EC, or both agents in combination, during 1 h of infection with RSV or SARS-CoV-2 Omicron, or MDCKII cells during 30 min of infection with IAV_PR8_. Next, the medium was changed, and cells were cultivated in the medium without interventions. For SARS-CoV-2 and IAV, cell culture supernatants were collected at three and two dpi, respectively, and virus production was quantified by qRT-PCR (Figure 7A,B). Regarding RSV, intracellular immunostaining was performed 2 dpi (Figure 7C).

Mono-treatment with 1 µg/mL IC or 1 µg/mL EC had only minor effects on the replication of SARS-CoV-2, and 0.5 µg/mL EC had no significant antiviral effect (Figure 7A, left side). However, combinatory treatment with low-dose combinations, 1 µg/mL IC and 0.5 µg/mL, significantly reduced virus replication by 50%, and 1 µg/mL IC together with 1 µg/mL EC by 70% (Figure 7A, left side), which indicates synergistic antiviral activity. This effect was even more pronounced for high-dose combinations of IC and EC (Figure 7A, right side). The combinatory treatment with 10 µg/mL IC and 10 µg/mL EC reduced the replication of SARS-CoV-2 Omicron by 90% (Figure 7A, right side).

Similar observations were made following infection with IAV. Treatment with 1 µg/mL IC or 0.5 µg/mL EC alone had only minor effects on the IAV replication. However, the combinatory treatment in this concentration range reduced the virus replication by 70% (Figure 7B, left side). The combinatory treatment with higher concentrations of IC and EC (both 10 µg/mL) almost completely inhibited viral replication (Figure 7B, right side), whereas the single treatment in this concentration range led only to a ~50% reduction. These results demonstrate that the combinatory treatment also blocks the replication of IAV in a synergistic manner.

For RSV, mono-treatment with 1 µg/mL IC reduced the replication by ~50%, whereas 0.5 µg/mL EC had no significant effect (Figure 7C, left side). However, the combinatory treatment with 1 µg/mL IC and 0.5 µg/mL EC almost completely blocked virus replication, which points towards a strong synergistic effect (Figure 7C, left side). Treatment with 1 µg/mL IC and 1 µg/mL EC inhibits the replication of RSV to undetectable levels, underscoring the synergistic activity of these two natural substances. The combinatory treatment with 10 µg/mL IC and/or 10 µg/mL EC exhibits an even more pronounced synergistic activity and also results in a complete block of RSV replication (Figure 7C, right side).

The cumulative data clearly show that the combinatory treatment with EC and IC derived from the same lozenges exhibits strong synergistic antiviral activity against SARS-CoV-2, IAV, and RSV when applied under co-treatment conditions.

To exclude that lozenge-derived EC and IC, as monotherapy or in combination, exhibit any nonspecific effects on cell viability, neutral red assays were conducted in uninfected Calu-3, MDCKII, or HEp-2 cells. The cells were treated for three (Calu-3) or two (MDCKII and HEp-2) days with the indicated concentrations of EC or IC alone or in combination (Figure 8). Staurosporine (10 µM) served as a positive control. The results clearly show that the treatment of all cell lines used in this study with IC and EC as mono- and combinatory therapy shows no nonspecific cytotoxic effects under any tested concentrations (Figure 8A–C).

## 4. Discussion

The term “*acute respiratory viral infections*” summarizes the most widespread illness in human populations, christened for centuries as the common “cold”, caused by almost all known respiratory viruses [57]. Thereby, especially respiratory RNA viruses remain a persistent driver of global morbidity, mortality, and socioeconomic disruption. Furthermore, it remains highly plausible that novel variants with pandemic potential will emerge in the future, as previously observed for SARS-CoV-1, MERS, and SARS-CoV-2, and during historic IAV pandemics, including the Spanish, Asian, Hong Kong, and Russian influenza. A major driver of this persistent threat is the evolutionary plasticity of RNA viruses: coronaviruses, orthomyxoviruses, and pneumoviruses (including RSV) rely on error-prone viral RNA-dependent RNA polymerases. In addition, segmented genomes such as IAV can additionally diversify via genome reassortment, collectively accelerating viral evolution. The ongoing co-circulation of major respiratory pathogens—together with ongoing zoonotic spillover risk—underscores the need for countermeasures that can be rapidly deployed and that ideally retain activity across virus families and variants [13,58,59,60].

Despite major progress in antiviral drug development, currently available direct-acting antivirals against SARS-CoV-2 and IAV still face clinically relevant constraints: their effectiveness depends strongly on early administration after first symptom onset; treatment failures can be driven by host factors and viral susceptibility; and some agents are limited by drug–drug interactions, contraindications, or restricted global availability [4,61]. For RSV, the prophylactic landscape has recently expanded—most prominently through licensed vaccines for older adults and maternal immunization strategies—yet small-molecule treatment options remain limited, and the development of effective, broadly accessible RSV therapeutics continues to be an active area of research [13,60]. Collectively, these realities support continued exploration of scalable, topical antivirals that act at the primary infection site.

Natural products and polymer-based antivirals represent an attractive complementary strategy because they can combine broad antiviral activity with favorable tolerability profiles. When their dominant mechanism targets conserved host–virus interaction principles, the barrier to resistance is comparatively high [58,59,62,63]. In recent years, numerous natural products have been reported to display antiviral activity in vitro and in vivo, and some candidates have progressed into clinical studies [59,62,63].

Here, we provide the first evidence that in vitro EC and IC cause a synergistic antiviral effect against SARS-CoV-2, IAV, and RSV without compromising cell viability. The observation of synergy across three phylogenetically and structurally distinct respiratory viruses suggests complementary mechanisms that may generalize beyond a single viral target. These results suggest that such a combinatory treatment could represent a broadly applicable option against multiple respiratory RNA viruses, including newly emerging variants. Moreover, our findings support EC/IC as promising candidates for broadly active topical antiviral formulations aimed at early infection stages.

Plant-derived juices and fruit extracts have repeatedly been identified as rich sources of antiviral bioactives. Extracts from berries and polyphenol-rich fruits (including blackberry, blackcurrant, mulberry, and pomegranate) have been reported to inhibit diverse viruses in vitro, spanning flaviviruses, orthomyxoviruses, coronaviruses, and retroviruses [64]. Such breadth supports the concept that complex phytochemical mixtures can interfere with infection through multi-target effects and/or virus particle-directed mechanisms. Over the last three decades, the successful use of *Sambucus nigra* (European black elderberry) has been supported by several clinical trials reporting that elderberry fruit preparations can reduce both the duration and severity of upper respiratory infections [19,20,65]. In parallel, multiple in vitro studies have aimed to clarify which molecular and cellular mechanisms may underlie the antiviral properties of black elderberry. Three main, non-mutually exclusive hypotheses have emerged from this body of work: (i) direct inhibition of viral infection steps by polyphenols (e.g., flavonoids and phenolic acids), including interference with attachment/entry and post-entry processes [54,66,67]; (ii) inhibition of viral enzymes required for efficient replication and spread (for IAV, neuraminidase inhibition has been described for specific constituents) [68,69]; and (iii) immunomodulatory activity of elderberry-derived polysaccharides that may modulate innate and adaptive host responses [70].

Beyond these established concepts, our data indicate a measurable extracellular component of EC activity, evidenced by reduced infectivity after pre-exposure of virions prior to cell contact. For influenza, elderberry-derived flavonoids have been reported to bind viral particles and impair hemagglutination, consistent with interactions at the level of surface glycoproteins [65,66]. More generally, polyphenols can bind to multiple sites on viral particles and promote virion clustering and stabilization, thereby reducing cell binding and infectivity [71]. In the present context, multivalent interactions between EC polyphenols and envelope glycoproteins (e.g., SARS-CoV-2 spike, influenza hemagglutinin, and RSV F/G) could mask receptor-binding surfaces, interfere with fusion processes, and/or drive aggregation of virions. These interpretations still remain hypothetical as long as further studies distinguish between virucidal inactivation of extracellular virions from interference of virions with attachment, fusion, or entry, as well as the following intracellular virus replication steps.

To analyze the influence of pre-exposure of virions to EC or IC, we established a protocol where we treated highly concentrated virus stocks with several inhibitor concentrations, followed by several log-dilution steps. Previously, we failed to purify virions following various purification steps like cushion centrifugation, as in all cases, the treated virions lost their infectivity nonspecifically. In the present pre-treatment protocol, we tested that due to several dilution steps, the remaining residual amounts of IC or EC during the infection were negligible. Regarding SARS-CoV-2, the IC_50_ values in the co-treatment setting were approximately 1:200 of EC and 10 µg/mL for IC (Figure 1B). In contrast, the residual amounts of EC or IC following infection with the pretreated virions were 2.18 log-fold lower than the calculated EC_50_ values during the infection period. Thus, the inhibition of virus replication following pre-treatment of virions must have been caused by a direct virucidal effect of the inhibitors on the virus particles. The same applies to the residual concentrations of the inhibitors that were potentially carried over with pretreated IAV or RSV virions. In addition, our conclusion that inhibitor concentration in the pretreated virus stocks following dilution steps would be way below any antiviral effective concentrations was further supported by the fact that the pre-treatment of IAV virions with the neuraminidase inhibitor Oseltamivir had no influence on virus replication in this time of addition setting (Figure 2A). This observation coincides with the mode of action of Oseltamivir, acting on the host cell but not on the virus particle [46].

Interestingly, the magnitude of inhibition depended on the type of virus and the time-of-addition regimen, with RSV showing the most pronounced susceptibility, particularly under pre- and co-treatment conditions. In particular, following mono-treatment with 10 µg/mL IC derived from lozenges, a very strong inhibitory effect on RSV replication was detectable (reduction of ~90%, Figure 7C). Therefore, in this setting of a high-dose combination of 10 µg/mL IC and 5 µg/mL EC, RSV replication was reduced to undetectable levels (Figure 7C). Altogether, this would be consistent with the idea that both natural inhibitors, EC and IC, either under mono- or combination treatment, engage virus-specific entry, attachment, or replication determinants, rather than producing a uniform ‘one-size-fits-all’ antiviral effect. The different time of addition experiments allows us to analyze the influence of the treatment with EC and/or IC on different steps of the virus replication cycle. Thereby, the pre-treatment of virions provides the first hint if the inhibitors exhibit direct virucidal activity, whereas the co-treatment rather analyzes the viral entry steps. Post-treatment over two or three days of infection illuminates the influence on the complete replication cycle, including the interference with intracellular replication, as in this time frame, multiple rounds of infection events occur. In addition, one should keep in mind that the extracellular virucidal effect applies to all times of addition settings, as it not only interferes with input virus (pre-treatment) but also with newly produced virions during co- and post-treatment.

Lozenges as well as nasal and throat sprays containing IC are certified for the prevention and treatment of the common cold and were sold in more than 30 countries all over the world [55]. The clinical benefit against respiratory cold viruses has been proven in multiple clinical trials and was undermined by an independent meta-analysis [56,72,73,74,75,76,77]. Most importantly, a placebo-controlled, double-blinded, and multicenter clinical study reported that an IC-containing nasal spray confers prophylactic efficacy against SARS-CoV-2 infection in healthcare personnel working in COVID-19 stations, with a relative risk reduction of 79.8% [35]. An earlier trial evaluating a nasal spray combining ivermectin and IC suggested reductions in viral loads as well as COVID-19 disease severity [34]. IC also has a favorable safety and regulatory profile that supports its use as a locally acting antiviral barrier. Both EFSA and the U.S. FDA have evaluated IC as a food additive, and the amounts administered via nasal/throat products are orders of magnitude lower than typical dietary exposure limits and are approved as food safe up to a quantum satis of 75 mg/kg body weight per day (i.e., 4500 mg/day for a 60 kg person) [78]. Consistent with this wide margin, high-molecular-weight IC has shown no immunotoxicity and no clinically local intolerance after intranasal application or inhalation in preclinical and clinical evaluations [26,79]. Across clinical trials of IC-containing nasal sprays, reported adverse events were generally comparable to saline controls [73,77]. Furthermore, iota-, kappa-, and lambda-carrageenan have been extensively evaluated in many animal studies (for review, see [79]). Thereby, they were administered orally, dermally, and via inhalation in quantities that are significantly higher than the concentrations of IC present in over-the-counter lozenges as well as nasal and throat sprays [79].

Mechanistically, IC activity is generally attributed to a physical mode of action [33,36]. The prevailing model is that IC forms a mucoadhesive and viscous layer on mucosal surfaces in which incoming particles are trapped and later removed by mucociliary clearance, while newly released virions may be captured as well, thereby reducing the effective viral load at the primary infection site [53]. At the particle level, the dense sulfate groups of IC confer a strong negative charge, enabling multivalent electrostatic interactions with positively charged regions on viral surface proteins and thereby reducing access to attachment interfaces; this can also promote virion clustering and immobilization within a polymeric network. Given the broad activity of IC against fundamentally different viruses, the trapping process is expected to be largely receptor-independent and predominantly physical.

RSV and related pneumoviruses/paramyxoviruses provide an additional mechanistic rationale for sulfated polysaccharides. RSV glycoproteins (F and G) can interact with sulfated glycosaminoglycans such as heparan sulfate/heparin, and competitive inhibition of these interactions by sulfated polysaccharides has been proposed as an antiviral strategy [80]. In support of this broader principle, IC has shown potent inhibition of human metapneumovirus (HMPV)—a clinically important respiratory virus closely related to RSV—by blocking heparan sulfate–dependent binding and infection in both cell culture and airway tissue models [81].

For IAV and SARS-CoV-2, it has been reported that chemically synthesized antivirals exhibit synergistic activity [82]. For example, for IAV, combinational treatment of favipiravir (a viral RNA polymerase inhibitor) and Oseltamivir (a neuraminidase inhibitor) was tested. Thereby, these two drugs showed synergistic antiviral activity, most probably because they block different stages within the replication cycle of IAV [83]. For SARS-CoV-2, promising synergy was reported for the polymerase inhibitor molnupiravir in combination with tilorone, an interferon inducer [84]. Regarding natural products, synergistic antiviral activity has previously been reported for combinations of different seaweed-derived extracts tested against the measles virus [83]. In addition, we recently demonstrated that EC in combination with quinine exerts synergistic antiviral activity against SARS-CoV-2 and IAV [23]. However, reports describing pronounced antiviral synergy between two defined natural products remain scarce. The synergy observed for EC/IC (Figure 4, Figure 5 and Figure 7) is most likely explained by complementary effects on early, extracellular infection determinants. IC can reduce the number of infectious particles to susceptible epithelial cells by trapping and adsorption within a mucosal barrier [33,53]. EC appears capable of decreasing virion infectivity directly (this work; [65,66,71]), lowering the number of particles that remain competent for attachment and fusion even if they reach the cell surface. In combination, EC-mediated masking and/or aggregation may increase retention of particles within the carrageenan network, while the mucoadhesive properties of IC can prolong local contact time and limit diffusion of infectious particles. However, these hypotheses regarding the mechanistic mode of action have to be proven in further studies. Together, these complementary effects could result in a more efficient blockade of virus attachment/entry than either component alone. Importantly, synergy was also observed when EC/IC was delivered in a lozenge-based format (Figure 7), supporting the feasibility of localized application at the upper respiratory tract.

Given that IC is already used in commercial nasal and throat sprays as well as lozenges [55,56], the present results may provide a direct basis for developing combination products. Leveraging the complementary mechanisms of EC and IC, the antiviral effect of the preparation would be intended to occur primarily in the oral cavity, particularly in the saliva and the mucosal tissues of the mouth. For IC, clinically achievable saliva levels after sucking IC-containing lozenges were previously shown in a clinical study to exceed the in vitro antiviral active range against SARS-CoV-2 by 60-fold [56]. Elderberry extract has also been clinically evaluated in a slow-dissolving lozenge formulation [85]. Data from this placebo-controlled pilot study revealed that EC-containing lozenges improved flu-like symptoms and were also well tolerated [85]. However, precise local oral exposure and residence time of EC remain to be defined. Nevertheless, our observation that lozenges containing both IC and EC exhibit synergistic antiviral activity in vitro, together with the previously conducted clinical studies, strongly suggests that the IC_50_ concentrations defined in our antiviral assays should be physiologically achievable. Formulations of these substances as lozenges or any other orally ingestible galenic forms, such as tablets, capsules, dragées, lozenges, or chewing gums, represent a simple, ready-to-use route of administration that would be broadly accessible over-the-counter.

Our studies are limited by the fact that results are derived solely from in vitro cell culture studies. Thus, the data obtained do not allow us to draw any definitive conclusions about the precise mode of action. Whether EC and/or IC act primarily virucidal, or if they also affect the target cell, or even exhibit multiple mechanisms of action, will be the subject of further investigations.

## 5. Conclusions

Taken together, the results of this study show that EC and IC synergistically inhibit three major respiratory RNA viruses in vitro by jointly targeting the earliest extracellular steps of infection through complementary particle-directed mechanisms. Notably, we provide the first experimental evidence of anti-RSV activity for EC and IC. The magnitude of inhibition was virus- and regimen-dependent, with RSV showing the strongest susceptibility, particularly under co-treatment. Finally, antiviral activity was maintained after incorporation into a lozenge matrix, supporting further mechanistic and translational studies to develop accessible prevention and early-treatment options.

## Data Availability

The original contributions presented in this study are included in the article. Further inquiries can be directed to the corresponding author.

## Abbreviations

The following abbreviations are used in this manuscript:

BSA	bovine serum albumin
Calu-3	human lung epithelial cell line
CaMg	calcium and magnesium
cDNA	complementary DNA
COVID-19	coronavirus disease 2019
DAPI	4′,6-diamidino-2-phenylindole
DMEM	Dulbecco’s Modified Eagle Medium
dpi	days post-infection
dsDNA	double-stranded DNA
EC	ElderCraft^®^ European black elderberry (*Sambucus nigra L.*) fruit extract
EFSA	European Food Safety Authority
FAM	6-carboxyfluorescein (fluorophore label)
FCS	fetal calf serum
FDA	U.S. Food and Drug Administration
GRAS	generally recognized as safe
HEp-2	human epithelial type 2 cell line
H&L	heavy and light chains
HMPV	human metapneumovirus
hpi	hours post-infection
IAV	Influenza A virus
IC	iota-carrageenan
IC_50_	50% inhibitory concentration (half maximal inhibitory concentration)
IgG	immunoglobulin G
kDa	Kilodalton
M2	Influenza A virus matrix protein 2 (ion channel)
MDCKII	Madin–Darby canine kidney II cells
MEM	Minimum Essential Medium
MERS	Middle East respiratory syndrome
MOI	multiplicity of infection
MTT	3-(4,5-dimethylthiazol-2-yl)-2,5-diphenyltetrazolium bromide
MW	molecular weight
NEAA	non-essential amino acids
NP	nucleocapsid protein
OSV	Oseltamivir
PBS	phosphate-buffered saline
PBS-T	phosphate-buffered saline with Tween
PFA	Paraformaldehyde
PFU	plaque-forming units
qRT-PCR	quantitative real-time reverse transcription PCR
RdRp	RNA-dependent RNA polymerase
RNA	ribonucleic acid
RPM	rounds per minute
RSV	respiratory syncytial virus
RT	room temperature
SARS-CoV-1	severe acute respiratory syndrome coronavirus 1
SARS-CoV-2	severe acute respiratory syndrome coronavirus 2
SD	standard deviation
STS	Staurosporine
VoCs	variants of concern
